# Computational approaches for identifying neuropeptides: A comprehensive review

**DOI:** 10.1016/j.omtn.2024.102409

**Published:** 2024-11-28

**Authors:** Roya Rahmani, Leila R. Kalankesh, Reza Ferdousi

**Affiliations:** 1 Student Research Committee, Tabriz University of Medical Science, Tabriz, Iran; 2 Department of Health Information Technology, School of Management and Medical Informatics, Tabriz University of Medical Sciences, Tabriz, Iran; 3 Tabriz University of Medical Sciences, Research Center of Psychiatry and Behavioral Sciences Tabriz, East Azerbaijan, Iran

**Keywords:** MT: Bioinformatics, neuropeptides, neuropeptide identification tools, neuropeptide databases, neuropeptide analysis

## Abstract

Neuropeptides (NPs) are key signaling molecules that interact with G protein-coupled receptors, influencing neuronal activities and developmental pathways, as well as the endocrine and immune systems. They are significant in disease contexts, offering potential therapeutic targets for conditions such as anxiety, neurological disorders, cardiovascular health, and diabetes. Understanding and detecting NPs is crucial because of their complex functions in health and disease. Historically, identifying NPs via wet lab techniques has been time-consuming and costly. However, integrating computational methods has shown the potential to improve efficiency, accuracy, and cost-effectiveness. Computational techniques, such as artificial intelligence and machine learning, have been extensively researched in recent years for the identification of NP. This review explores the application of machine learning (ML) techniques in predicting various aspects of NPs, including their sequences, cleavage sites, and precursors. Additionally, it provides insights into databases containing NP metadata and specialized tools used in this domain.

## Graphical abstract

**Figure:**
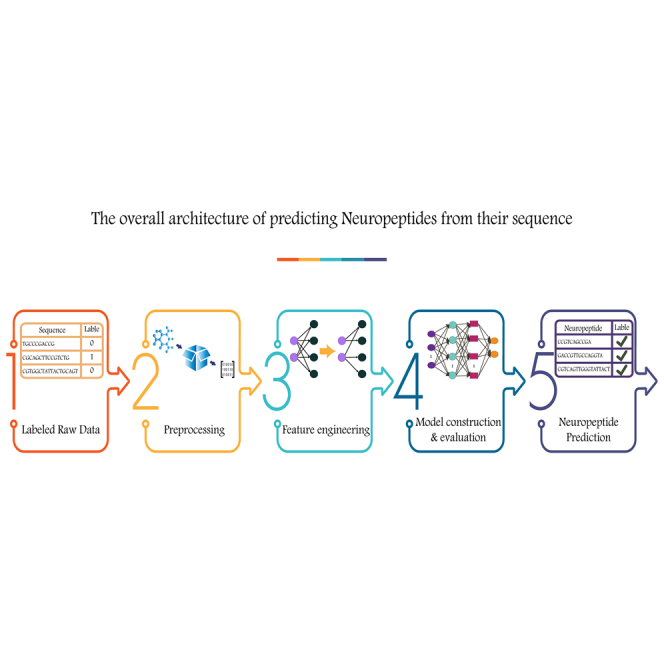

## Teaser

This review highlights neuropeptide (NP) prediction tools developed with artificial intelligence, emphasizing their value in supplementing wet lab methods for greater efficiency. Proven as active agents in the gut-brain axis, NPs hold therapeutic promise across neurological, cardiovascular, and endocrine domains, paving the way for targeted interventions.

## Introduction

Neuropeptides (NPs) represent a complex and extensive group of signaling molecules that interact with G protein-coupled receptors.^1^ These messenger molecules impact a wide range of neuronal activities and contribute to crucial developmental processes, including biological, cognitive, and socioemotional aspects.^2^ Although NPs are present in glial cells, they play multiple roles, such as acting as neurotransmitter peptides in the endocrine system and as hormonal peptides in the immune system.^3^ Given their diverse functions, NPs significantly influence muscle contraction, food digestion, and various physiological and behavioral processes such as learning, memory, adaptation, and aging.^4^ The diverse functionalities of NPs in living species contribute to their significant impact on the development and progression of various diseases and their amelioration and improvement. Consequently, NPs have emerged as promising therapeutic targets for a diverse range of diseases affecting various physiological systems, including anxiety, behavior,^5^^,^^6^ neurology,^7^ cardiovascular health,^8^ diabetes,^9^ and a multitude of other conditions.^10^

NP identification has received significant attention from scientists, leading to the development of different types of methods over the years to identify various types of peptides.

Corbière and colleagues comprehensively reviewed different approaches for identifying bioactive NPs in vertebrates.^11^ Their research covered various methods, including identification on the basis of biological activity, receptor analysis, the biochemical characteristics of the NPs, genomic approaches, peptidomics approaches, and *de novo* identification. While wet lab methods are extremely time-consuming and costly,^12^^,^^13^ the integration of computational approaches has significantly enhanced efficacy, accuracy, and cost-effectiveness.^14^^,^^15^

In alignment with the valuable contributions of Corbière et al.,^11^ this study explores the realm of computational approaches for NP prediction. By leveraging cutting-edge technologies, our research aims to provide an alternative and complementary viewpoint for identifying NPs.

Computational and *in silico* approaches have played a significant role in the majority of methods used for identifying NPs, complementing laboratory experiments. These methods include genomic-based peptide identification and mass spectrometry (MS), both of which utilize various computational techniques such as data analysis, bioinformatics, and predictive modeling. Genomics-based peptide identification relies heavily on computational analysis for tasks such as genetic sequence examination, sequence alignment, NP precursor (NPP) prediction, and conserved motif analysis.^16^ In the case of MS, computational algorithms and specialized software are used to reconstruct peptide sequences and predict peptide structures through *de novo* identification analysis.^13^^,^^17^ Additionally, computational methods are often employed to simplify statistical analysis, data interpretation, and result validation. The rise of AI and machine learning (ML) approaches in recent years has further enhanced peptide identification, particularly in predicting NP sequences.^13^^,^^18^^,^^19^

**Figure 1:**
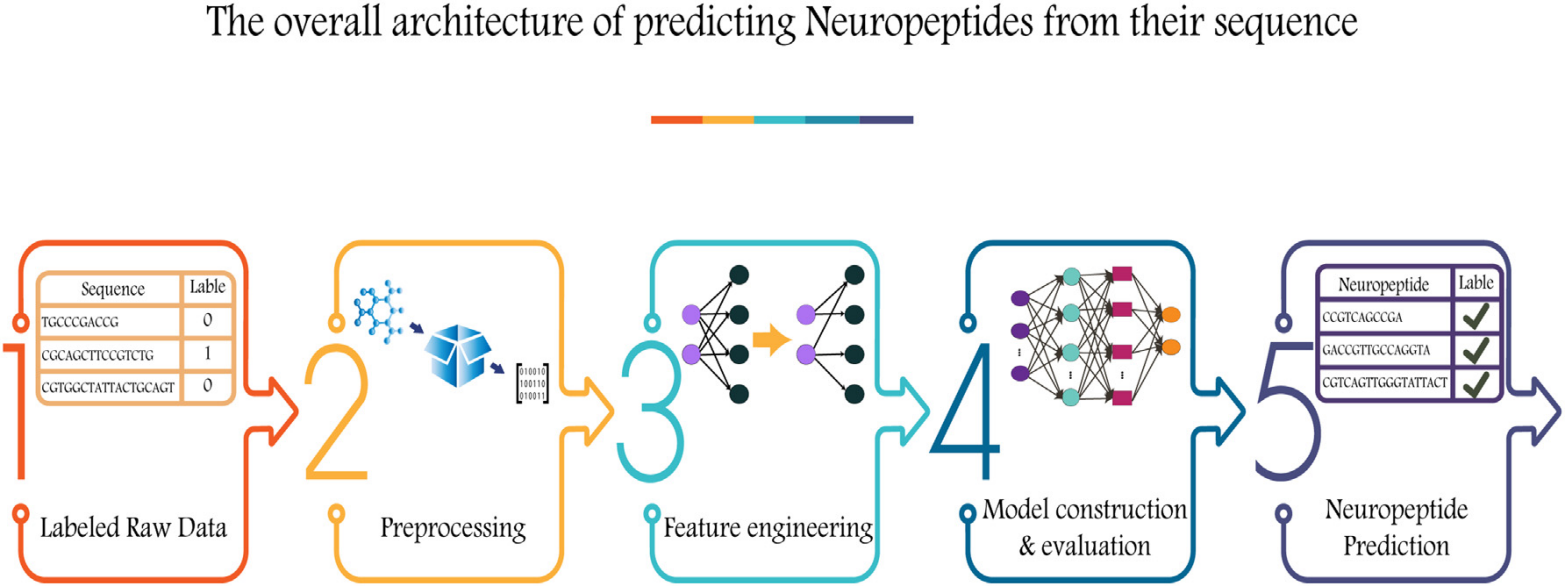
The overall architecture of NP prediction based on the literature A systematic representation of the NP prediction framework based on information from the literature. The diagram outlines key stages in the NP prediction process, including data input, feature extraction, model training, and output.

According to the literature, the overall architecture of NP prediction is presented in Figure 1. The process of predicting NPs through ML/deep learning (DL) techniques has been summarized in five distinct phases. Researchers extract labeled raw data from available databases. Next, the data are preprocessed for feature engineering. The methods utilized for feature engineering are classified into two groups: feature encoding schemes and feature embeddings, and these are summarized in Tables 1 and 2, respectively. In the next phase, feature importance algorithms are often used to optimize the feature matrix dimensions. The optimized features are subsequently fed into the constructed ML or DL model following evaluation, and once satisfactory accuracy is achieved, the model can be deployed to predict NPs from unknown sequences. Several studies have made tools available through web services or Python packages, facilitating further utilization by researchers in this field.

**Table 1:** Feature encoding schemes utilized by different tools

	Studies	Utilized feature encoding schemes
**Cleavage site predictor**		

1	NeuroCS	CTD DDE PAAC EAAC
2	NeuroPID	biophysical quantitative properties binary features information-based statistics

**NPP predictor**		

3	NeuroPP	AAC DPC TPC CPC OCPC

**NP predictor**		

4	Ridzik and Brejová	one-hot
5	NeuroPIpred	AAC Dipeptide composition (DPC) SC BP
6	PredNeuroP	AAC DPC BPNC AAI GAAC GDPC GTPC CTD AAE
7	NeuroPpred-Fuse	AAC DPC ASDC GGAP CTD PSAAC
8	NeuroPred-FRL	BE AAI KmerAC KgapAC PrAC TPPC) KgapAP GDPC GTPC QSO CTF
9	NeuroPpred-SVM	AAC PSAAC AAP AAT CTD
10	NeuroPred-CLQ	one-hot supervised embedding
11	NeuroCNN_DNB	one-hot AAI GGAP
13	Akbar et al.	Bi-PSSM KSB discrete wavelet transforms from Bi-PSSM_DWT and KSB_DWT

AAC, amino acid composition; AAE, amino acid entropy; AAI, amino acid index; AAP, amino acid pair scale; AAT, amino acid trimer scale; ASDC, adaptive skip dipeptide composition; BE, binary encoding; Bi-PSSM, bigram position-specific scoring matrices; BP, binary profile; BPNC, binary profiling feature; CPC, combined peptide composition; CTD, composition-transition-distribution; CTF, conjoint triad; DDE, dipeptide deviation from expected mean; DPC, dipeptide composition; DWT, discrete wavelet transform; EAAC, enhanced amino acid composition; GAAC, grouped amino acid composition; GDPC, grouped dipeptide composition; GGAP, G-gap dipeptide composition; GTPC, grouped tripeptide composition; KgapAC, K-gap composition of amino acids; KgapAP, K-gap composition of profile-based amino acids; KmerAC, Kmer composition of amino acids; KSB, K-spaced bigram; OCPC, optimal combined peptide composition; PAAC, pseudo-amino acid composition; PrAC, profile-based composition of amino acids; PSAAC, position-specific amino acid composition; QSO, quasi-sequence order; SC, split composition; TPC, tripeptide composition; TPPC, tripeptide profile of amino acid composition.

**Table 2:** Feature embeddings utilized by different tools

	Studies	Utilized feature embeddings
1	DeepNeuropePred	ESM (protein language model)
2	NeuroPpred-SVM	BERT-cls BERT-avg
3	NeuroPred-PLM	ESM (protein language model)
4	NeuroPred-CLQ	word2vec embedding
5	NeuroCNN_DNB	Skip-Gram

BERT, bidirectional encoder representation from transformer; SVM, support vector machine.

The following section explores computational research on NPs, focusing on methods for predicting NP sequences, cleavage sites, and precursors. The subsequent section discusses several databases containing NP metadata or specifically designed for NP-related data. The section following that provides an overview of the bioinformatics tools utilized in NP prediction models, databases, and tools.

### Computational research on NPs

Over the years, ML approaches have been implemented in three aspects of NP sequences, including predicting cleavage sites,^14^^,^^20^^,^^21^ predicting NPP identifiers,^22^^,^^23^^,^^24^ and predicting NPs from their sequences,^25^^,^^26^^,^^27^^,^^28^^,^^29^^,^^30^^,^^31^^,^^32^^,^^33^ which are clarified separately in Table 3.

**Table 3:** Computational approaches proposed for predicting three aspects of NPs

	Name	Type of prediction	Method (model with the best performance)	Recruited data	ACC	Sp	Sn	AUROC	MCC	Feature extraction	Base learner	Meta learner	Web server
**Neuropeptide cleavage site predictors**													

1	NeuroPred (2003–2008)^14^^,^^20^^,^^34^^,^^35^^,^^36^	NCS	Statistical (binary logistic regression analyses)	aplysia prohormones: literature	0.97	0.96	0.97	–	–	–	LR	–	✔
2	DeepNeuropePred (2024)^37^	NCS	DL (CNN)	NPPs: UniProt	0.87	–	–	0.78	0.65	ESM	CNN	–	–

**Neuropeptide precursor prediction**													

3	NeuroPID (2014)^22^^,^^23^	NPPI	ML (SVM and ensemble decision tree classifiers)	NPPs: UniProt	0.89–0.94	–	–	0.89–0.94	–	–	SVM, ensemble decision tree	–	✔
4	NeuroPP (2019)^24^	NPPI	ML (optimally combined peptide composition based-SVM)	NPPs: Uniport and NeuroPedia	0.886	–	–	0.95	0.78	OCPC	SVM	–	✔

**Neuropeptide prediction**													

5	Ridzik and Brejová (2014)^38^	NP	ML (SVM and semi-CRF)	NPPs: UniProt	0.704–0.791	–	–	–	–	–	SVM, semi-CRF SVM	–	X
6	NeuroPIpred (2019)^25^	NP	ML (DPC-SVM)	NPs: UniProt and SATPdb, non-NP: DINeR	DS1: 0.837 DS2: 0.974	DS1: 0.851 DS2: 0.974	DS1: 0.822 DS2: 0.982	DS1: 0.910 DS2: 0.990	DS1: 0.670 DS2: 960	DPC	SVM	–	✔
7	PredNeuroP (2020)^26^	NP	ML (BPNC- ANN)	NPs: NeuroPep, non-NPs: UniProt	0.872	0.900	0.886	0.957	0.787	BPNC	ANN	LR	X
8	NeuroPred-FRL (2021)^27^	NP	ML (BE-SVM-XGBoost)	NPs: NeuroPep	0.916	0.903	0.929	0.960	0.834	BE	SVM	XGBoost	X
9	NeuroPpred-Fuse (2021)^28^	NP	ML (relief-XGBoost)	NPs: NeuroPep, non-NPs: UniProt	0.906	0.958	0.930	0.813	0.958	AAC, DPC, ASDC, GGAP, CTD, PSAAC	Relief	XGBoost	✔
10	NeuroPred-CLQ (2022)^29^	NP	DL (CBOW-CNN-TCN-Mul)	NPs: NeuroPep, non-NPs: UniProt	0.936	0.975	0.897	0.988	0.875	word2vec (CBOW)	CNN-TCN-Mul	–	X
11	NeuroPpred-SVM (2023)^31^	NP	ML (BERT-avg-SVM	NPs: NeuroPep, non-NPs: UniProt	0.910	0.911	0.909	0.969	0.820	BERT-avg	SVM	–	X
12	NeuroPred-PLM (2023)^32^	NP	DL (ESM-CNN-Mul)	NPs: NeuroPep 2.0, non-NPs: UniProt	0.927	0.926	0.928	–	0.854	ESM	PLM-CNN-MUL	–	✔
13	NeuroCNN_GNB (2023)^30^	–	DL (Skip-Gram-CNN-Gaussian NB)	NPs: NeuroPep, non-NPs: UniProt	0.918	0.917	0.919	0.962	0.836	Skip-Gram	CNN	Gaussian NB	✔
14	Akbar et al. (2023)^33^	–	–	NPs: NeuroPep, non-NPs: UniProt	0.925	0.938	0.913	0.87	–	one-hot encoding, KSB, Bi-PSSM	XGBoost, ensemble-GA	–	–

Performance metrics are based on different validation datasets across studies and are provided for reference only, not for direct comparison across tools.

NCS, Neuropeptide cleavage site prediction; NPPI, neuropeptide precursor identifiers prediction; NP, neuropeptide prediction; ML, machine learning; DL, deep-learning; ANN, artificial neural network; CNN, convolutional neural network; Mul, multi-head attention; ACC, accuracy; Sp, specificity; Sn, sensitivity; AUROC, area under the receiver operating characteristic curve; MCC, Matthew’s correlation coefficient; GNB, Gaussian NB, Gaussian Naive Bayes; ensemble-GA, ensemble Genetic Algorithm; LR, Logistic regression.

Table 3 summarizes various approaches in NP prediction, providing details of each method. However, it should be pointed out that performance metrics such as accuracy, specificity, sensitivity, the area under the receiver operating characteristic curve, and the Matthews correlation coefficient are drawn from studies that may use different validation datasets. Therefore, these values should be viewed as reference points rather than as a direct comparison of tool performance. These data were collected from the published text of each article. While more software has been developed to predict other types of proteins for different purposes, this article focuses specifically on NP-related software published in the literature.

**Figure 2:**
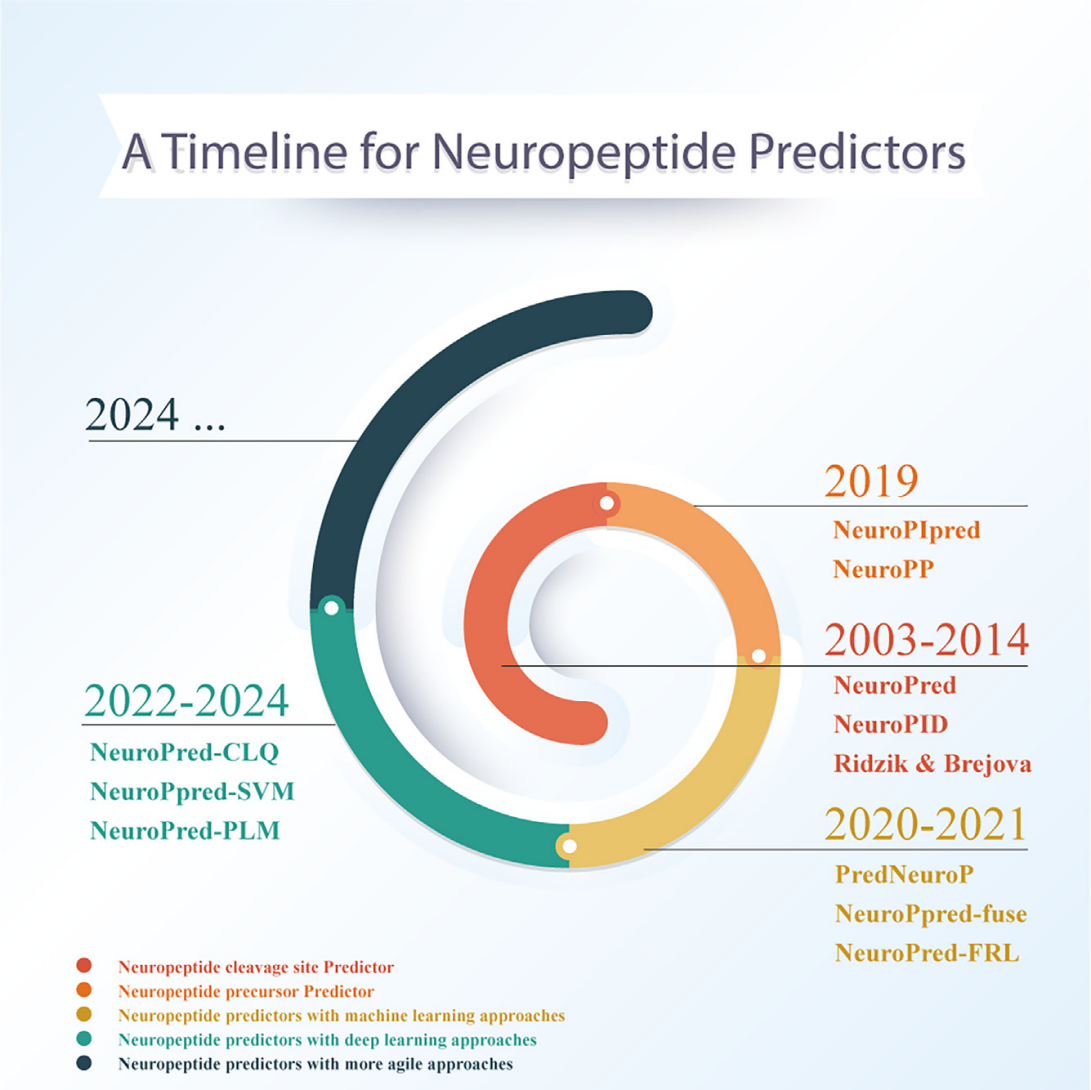
Development time line of NP prediction tools A chronological time line shows NP-related tool development with specific predictive focuses: neuropeptide predictors, neuropeptide cleavage site predictors, or neuropeptide precursor predictors. Each color highlights the year and main advancements in each predictive aspect.

With the available data in early 2003, researchers developed complicated strategies to predict NPs from their cleavage sites or their NPP identifiers. However, with the publication of more suitable data for ML/DL models in the field of NPs, researchers have advanced in the prediction of the amino acid sequence of NPs. Since 2014, 14 tools have been designed via this approach, which are discussed in this article. An overview of the tools developed for predicting different aspects of NPs is provided in Figure 2. This figure elucidates the time line and specific predictive functionality of each tool.

The following three subsections discuss the available tools developed over the years for predicting various aspects of NPs.

### Computational approaches for identifying NP cleavage sites

The prediction of the cleavage site of NPs is highly important in contemporary neurobiological research.^12^ NPs play diverse and critical roles in various brain systems and neuronal networks, exerting influence on development and behavior.^5^^,^^6^ However, their complex enzymatic processing system, involving the cleavage of long protein precursors and post-translational modifications, presents significant challenges in predicting these sites on the basis of precursor sequence information.^39^^,^^40^^,^^41^ Moreover, NPs often exhibit limited neuronal synthesis, temporal regulation during development, or low concentrations, complicating their experimental confirmation.^42^ Hence, the ability to predict cleavage sites becomes crucial for understanding the functional roles of NPs and elucidating their impact on neural processes.^34^

In recent years, researchers have made notable efforts to develop peptide cleavage site predictors, such as DeepPeptide,^43^ PeptideLocator,^44^ and NeuroCS.^21^ This section presents some of the efforts specifically focused on NP cleavage site prediction. For example, NeuroPred is a user-friendly Python-based web application designed to predict cleavage sites in NPPs and calculate the masses of resulting peptides. This tool has been the focus of five articles published from 2003 to 2008, all of which are dedicated to its design and evolution. Initial efforts by Hummon et al. involved developing statistical models to predict cleavage probabilities in mollusk (specifically *Aplysia californica*) prohormone data, exported from the literature, achieving 97% accuracy via logistic regression.^20^ Amare et al., from the same research team, extended this work to mammalian prohormones, achieving superior sensitivity (81.7%) compared with previous models.^35^ In their 2006 publication, the research team led by Bruce R. Southey expanded the prediction scope to RFamide peptides, employing logistic regression, neural networks, and motif-based approaches and achieving high predictive accuracy, with area under the receiver operating characteristic curve values up to 98%. These advancements culminated in the creation of NeuroPred, incorporating logistic regression.^34^ In 2008, the tool’s capabilities were further extended to insect NP prediction, demonstrating robust accuracy across diverse datasets.^36^ NeuroPred remains an essential resource in NP research, as evidenced by its application in recent studies, including the identification of uncharacterized NPs in American lobsters by Lu et al.^45^ Moreover, a growing body of literature has employed NeuroPred to explore NP processing in various organisms, underscoring its importance in advancing neurobiological research.^46^^,^^47^

In 2005, during the process of developing NeuroPred, Liu and Wets developed a neural network method to predict proteolytic cleavage sites in NPs.^48^ Initially, known cleavage sites from vertebrate NPPs annotated in the Swiss-Prot database^49^ were identified. Following data cleaning and encoding, the class imbalance were addressed via SMOTE (synthetic minority over-sampling technique)^50^ combined with Tomek links^51^ for oversampling. After the data were balanced, one-hot encoding was used for the neural network model. The neural network trained with the backpropagation algorithm demonstrated high sensitivities ranging from 79% to 91% for various cleavage sites. Performance was evaluated through cross-validation, ensuring robust predictions. This model not only accurately identified consensus-matching cleaved sites but also predicted other cleavage sites not recognized by earlier rules, representing substantial advancements in the field.

In recent years, Wang et al. have drawn on insights from NeuroPred and other tools developed for peptide cleavage sites of various proteins. DeepNeuropePred was developed specifically to predict NP cleavage sites.^52^ Limitations of existing models, such as limited datasets and simplistic representation models, were addressed by combining pre-trained language models and convolutional neural networks (CNNs) to extract features and predict NP cleavage sites. Initially, a dataset was compiled from the UniProt/KB database^49^ consisting of 1,194 completely reviewed NPPs, which were filtered down to 717 precursors to ensure fairness in comparisons with the independent test dataset. Next, a transformer-based self-supervised language model called ESM^53^ was utilized to extract the global feature representation of the NPPs. The feature importance analysis was then implicitly performed via the ESM model and multiscale CNNs to ensure that the relevant features were utilized for accurate predictions. The researchers constructed the DeepNeuropePred model and evaluated it against four models from the NeuroPred server via an independent dataset. The results indicated that DeepNeuropePred achieved the highest accuracy of 87%. Finally, the model was made available through a user-friendly web server, providing a valuable tool for researchers in the field.

Together, these advancements highlight the significant progress made in predicting NP cleavage sites over the past decade. The evolution from statistical models to neural networks and advanced ML techniques, such as those employed in NeuroPred and DeepNeuropePred, underscores the increasing sophistication and accuracy of these tools. These tools not only facilitate the discovery and characterization of NPs but they also pave the way for future research and potential clinical applications in neurobiology.

### Computational approaches for NPP identification

Predicting NPPs is important for understanding the intricate functions orchestrated by NPs across metazoan life. NPs, which are pivotal in numerous physiological and behavioral processes, act through specific receptors, initiating signaling cascades that modulate various cellular activities.^1^ NPPs serve as reservoirs for multiple NPs, with cleavage events at specific motifs, typically characterized by basic residues such as Arg and Lys, leading to the generation of mature peptides.^54^ Despite their critical roles, NPPs are often challenging to identify, especially in poorly annotated metazoan genomes.^46^ The discovery and characterization of NPPs are particularly crucial for drug development and pharmacological interventions, given their involvement in various physiological processes and potential as therapeutic targets.^55^ This section delves into the efforts of researchers in this field, exploring the advancements and challenges in NPP prediction tools.

Ofer and Linial innovated NeuroPID, an innovative ML framework tailored for predicting NPP identifiers.^22^ Initially, data were gathered and curated from the UniProtKB database,^49^ focusing on annotated NPPs while excluding NP receptors and incomplete sequences. Next, features from the primary sequences of known NPPs were compiled, encompassing biophysical quantitative properties, binary features, and information-based statistics (Table 1). For feature engineering, statistical methods such as two-sample Kolmogorov-Smirnov tests and t tests were applied to compare the distributions of these features between positive (NPPs) and negative (non-NPP) sets. A support vector machine (SVM) and an ensemble decision tree classifier were then used to process these features, achieving high accuracy ranging from 89% to 94% in cross-validation tests. The capabilities of NeuroPID were later extended by Karsenty et al. from the same research team to identify NPPs from unannotated transcriptomes and MS experiments.^23^ Using the same ML models as in the original framework, the enhancements maintained comparable accuracy and precision levels of 89%–94% and 90%–93%, respectively, in the cross-validation test. A user-friendly web tool for NeuroPID was created to make the model more accessible and is available online.

Another application aimed at NPP prediction is NeuroPP, a publicly available user-friendly web server.^24^ The input data were collected from the Swiss-Prot^49^ and NeuroPedia^56^ databases. Next, five different kinds of features were extracted from the input data, including various peptide compositions, as detailed in Table 1. The combined peptide compositions were further refined to create an optimized feature set. Five ML models were constructed using an SVM algorithm implemented via LibSVM software.^38^ These models were trained using 5-fold cross-validation using different feature sets. The model utilizing the optimal combined peptide composition feature set achieved the best performance, with an accuracy of 95%. Finally, the resulting tool, NeuroPP, is made available as a user-friendly web server.

### Computational approaches for NP identification

Since 2014, the trend in NP prediction has shifted toward predicting NPs from amino acid sequences. Earlier methods predominantly utilized handcrafted feature extraction techniques (Table 1), whereas recent publications have increasingly emphasized the development of DL models, necessitating more efficient methods for feature embeddings. This section explores various tools developed for NP prediction.

The development of NP prediction software began in 2014 when Ridzik and Brejová initiated efforts to identify NPs. Critical challenges were addressed in their research such as identifying precursor proteins, predicting cleavage sites within these precursors, and annotating NPs derived from precursor proteins.^57^ External software, SignalP,^58^ was used to identify NPPs, alongside two prediction models: an SVM model to predict cleavage sites in NPPs and a semi-conditional random field (semi-CRF) model to annotate the NP sequences. Despite the existence of NeuroPred as a known tool for NP cleavage site prediction at the time, researchers opted to develop an independent NP cleavage site prediction tool due to NeuroPred’s limitation as a web-based service, which hindered its integration into a stand-alone tool.

To develop the SVM model, known NPPs from Arthropoda and Metazoa were extracted from the UniProt database.^49^ Next, amino acids were encoded into binary vectors within sequence windows, focusing on those containing lysine or arginine residues preceding the cleavage site. Features were further refined by selecting an 11-amino acid window size and applying the radial basis function kernel for a non-linear discriminant function, enhancing precision and ensuring fast convergence. The SVM model was implemented using the LibSVM library.^38^ Through cross-validation, the model reached a classification accuracy of 91.1% on the testing data, slightly outperforming NeuroPred.

Following this, the output sequences from SignalP^58^ and the cleavage site predictor SVM model were fed into the NP annotation model to label or annotate regions within proteins as NPs based on specific characteristics. First, the annotated protein sequences were extracted from the UniProt database.^49^ Second, proteins were clustered based on sequence similarity with the CD-HIT tool, and sequences with incomplete or ambiguous annotations were filtered out. Third, a semi-CRF model was employed to annotate the NPs. In addition to the modeling efforts, feature selection algorithms were applied to increase prediction accuracy, likely considering various aspects of protein sequences such as specific patterns or characteristics common to NPs.

The cleavage site prediction and the NP annotation models were integrated into a larger framework. Given a protein sequence as input, the system first utilized the cleavage site prediction model to identify potential cleavage sites within the precursor protein. These predicted cleavage sites were then used to segment the protein sequence into putative NPs. The NP annotation model subsequently evaluated each segment to determine whether it exhibited characteristics indicative of an NP. Segments that were positively labeled by the annotation model were considered to be predicted NPs. Finally, both models were evaluated using the same data, and their system was able to accurately recognize 79.1% of the NP sequences.

In 2019, Piyush Agrawal and coworkers developed NeuroPIpred, a user-friendly web application aimed at predicting, designing, and scanning insect NPs.^25^ For training purposes, two distinct datasets were constructed: NeuroPIpred_DS1, containing natural NPs, and NeuroPIpred_DS2, for C-terminal amidated NPs. Negative data were extracted from Swiss-Prot and SATPdb^59^ (structurally annotated therapeutic peptides), and positive data were drawn from DINeR^60^ (database for insect NP research). Next, amino acid composition and positional residue preference analyses were conducted to identify key residue preferences in insect NPs, employing four distinct feature types clarified in Table 1. To reach the best combination of features and classifiers, various feature sets were trained with different kinds of ML models consisting of SVM, random forest (RF), naive Bayes, J48, and SMO, through WEKA classifiers.^61^ WEKA (Waikato Environment for Knowledge Analysis) is an open-source machine learning software suite that supports a range of classification, regression, clustering, and data preprocessing tasks. Among the tested models, an SVM model based on the dipeptide composition feature set achieved the best performance. Model accuracy varied by dataset, reaching 83.71% for NeuroPIpred_DS1 and 97.93% for NeuroPIpred_DS2.

To improve model performance, Bin et al. developed PredNeuroP,^26^ a two-layer stacking predictor, to distinguish NP sequences from non-neuropeptide (non-NP) sequences. Positive data, consisting of NPs with fewer than 100 residues, were extracted from the NeuroPep database.^62^ Owing to the lack of experimentally validated non-NPs, negative data were sourced from Swiss-Prot via the query “NOT neuropeptide AND sequence length range 5 to 100” following criteria from previous studies.^37^^,^^63^ Next, nine different feature descriptors were employed, including composition-based, binary profile-based, physicochemical property-based, and position-based features, as detailed in Table 1. PredNeuroP utilized a two-layer stacking framework. In the first layer, 45 models are trained by combining 9 feature descriptors with 5 ML algorithms (artificial neural network [ANN], extremely randomized tree [ERT], K-nearest neighbor [KNN], logistic regression, and extreme gradient boosting [XGBoost]). Based on statistical criteria, only the top eight models advanced to the second layer, where their outputs were fed into a logistic regression classifier, which generated the final NP prediction. This integration of various models and feature encodings enabled PredNeuroP to accurately predict NPs from non-NPs. After 10-fold cross-validation, final accuracies of 89.3% and 87.2% were achieved on the training and test datasets, respectively. PredNeuroP was subsequently deployed as an NP prediction command line tool, which is available as a Python library for installation on Windows and Linux.

Building on these advancements, Hasan et al. developed NeuroPred-FRL, a web server that leverages ML algorithms and feature representation learning (FRL) to predict NPs.^27^ The same database as in the Bin et al. study was used, along with similar preprocessing steps. Eleven diverse feature encoding schemes were applied to capture various aspects of peptide sequences, as detailed in Table 1. Six distinct ML classification algorithms, including ERT, KNN, naive Bayes, AdaBoost, and SVM, were employed, culminating in 66 baseline models. Evaluation of these models showed that the combination of binary encoding and the SVM algorithm achieved the highest accuracy of 91.9%. Compared with PredNeuroP, NeuroPred-FRL demonstrated a 2.3% greater performance, attributed to the adoption of an FRL approach over the single feature-based approach used by PredNeuroP. NeuroPred-FRL was ultimately deployed as a user-friendly web tool, allowing users to input sequences or fasta files and receive detailed result interpretations.

By leveraging a combination of feature selection and ensemble learning, Jiang et al. developed NeuroPpred-Fuse, an interpretable two-layer stacking model for identifying NPs based on their sequence information.^28^ The same data as in the Bin et al.^26^ study was utilized, with similar filtering methods applied. Six feature encoders were employed to extract various features from the sequences, which were then fused into a single input feature vector. To reduce the dimensionality of the input features, four feature selection algorithms were applied—Relief, Boruta, variance-based selection, and F-score-based selection. These algorithms acted as filters to prioritize the features that were the most significant to NP identification. The filtered features were then fed into three base learners: random forest, gradient boosting decision tree, and XGBoost. These learners built individual models to predict the presence of NPs, with each model capturing different aspects of the data. The first layer of the model involved combining the predictions from all three base learners to leverage their complementary strengths. The second layer of NeuroPpred-Fuse aggregated the outputs from the first layer and fed them into a logistic regression classifier, which serves as a meta-learner to integrate the knowledge from the first layer and make a final prediction on whether a given sequence represents an NP. Using 10-fold cross-validation, the authors reported an accuracy of 90.6% for the best model configuration, which utilized Relief for feature selection and XGBoost as the base learner.

As the NP prediction models have become more popular, Chen et al. ingeniously crafted NeuroPred_CLQ, a DL model for NP prediction.^29^ The same datasets from the NeuroPep and Swiss-Prot databases, as in PredNeuroP by Bin et al.^29^ were employed, ensuring consistency in data sources. An innovative feature representation approach was implemented using word2vec, a two-layer neural network embedding technique widely used in natural language processing. Using the continuous “bag_of_words” (CBOW) technique implemented in the Python Gensim library,^64^ textual words were converted into numerical vectors. A multihead attention mechanism was applied to extract salient features and improve model stability. This attention mechanism allows the model to weigh different parts of the input sequence differently, capturing more nuanced information. Among various hybrid models tested, a combination of a CNN, a temporal convolutional network (TCN), and multihead attention achieved the highest accuracy of 93.6%. This hybrid approach effectively integrates the strengths of different neural network architectures to improve prediction performance. NeuroPred_CLQ was subsequently packaged as an installable Python tool, facilitating easy deployment for NP prediction.

Expanding on these methodologies, Liu et al. developed NeuroPpred-SVM, a web server to predict NPs, which was constructed via a two-layer ML model.^31^ Data extracted from the NeuroPep and Swiss-Prot databases and the preprocessing methods used by Bin et al.^26^ were utilized to prepare the data. To enhance prediction accuracy, a diverse pool of feature representations was leveraged. Seven feature encoders and 14 learning algorithms, resulting in 90 distinct models were employed in the first layer of NeuroPpred-SVM.^31^ This layer aimed to capture various aspects of NP sequence information. Feature importance analyses were performed, where the model explored the different representations to select the most significant features for NP prediction. The second layer of the model was constructed by implementing a meta-learner to integrate the knowledge from the first layer. This meta-learner was built via two algorithms: SVM and logistic regression. The SVM-based model outperformed the logistic regression model, suggesting its superior ability to learn from diverse first-layer predictions. The software was built by combining the bidirectional encoder representation from transformer (BERT) embeddings with three specific feature encoding schemes (composition-transition-distribution [CTD], position-specific amino acid composition [PSAAC], amino acid trimer scale [AAT]) and utilizing the SVM algorithm. This final model has an estimated accuracy of 91.5%, demonstrating its competitive performance in NP prediction. The software was made available as an accessible user-friendly web server, facilitating practical application in NP prediction.

Continuing the trend of advancing NP prediction methodologies, Wang et al. developed NeuroPred-PLM, a user-friendly software for NP prediction.^32^ A comprehensive dataset of 11,282 experimentally validated NP sequences from NeuroPep 2.0^65^ was compiled, while non-NP sequences were gathered from Swiss-Prot using methodologies similar to those used by Bin et al.^26^ for negative dataset selection. The preprocessing and filtering procedures closely mirrored those of Bin et al.,^26^ with the exception that unnatural amino acids were not excluded from the dataset in NeuroPred-PLM. For feature extraction, the approach employs a 12-layer protein language model (ESM) combined with a multiscale CNN, which effectively captures complex sequence features. A global multihead attention network was incorporated, which enhanced the ability of the model to identify the most relevant features and improve interoperability and performance. The model was constructed and evaluated on an independent test set, employing a 5-fold cross-validation that demonstrated an average accuracy of 92.7%. NeuroPred-PLM^32^ was developed into a user-friendly software package with a convenient web interface and straightforward installation via the PyPi package,^66^ facilitating widespread adoption and practical application in NP prediction.

Continuing the trajectory of advanced NP prediction methodologies, Liu et al. proposed NeuroCNN_GNB, a web-accessible two-layered stacking model for NP prediction.^30^ A dataset akin to that of Bin et al., sourced from NeuroPep and Swiss-Prot, was utilized, ensuring alignment with established practices in NP research. Preprocessing filters, identical to those of Bin et al.,^26^ were applied, refining the dataset. Information from filtered NP sequences was obtained via four different encoding schemes: one-hot encoding, physicochemical-based features, amino acid frequency-based features, and word2vec embeddings. These extracted features underwent optimization via a grid search within a CNN framework. The model construction leveraged a stacking ensemble strategy that integrated CNN models tailored to various feature representations, including Skip-Gram embeddings. The combination of Skip-Gram embeddings and Gaussian Naive Bayes (NB) achieved the highest accuracy of 91% by 5-fold cross-validation. Finally, a user-friendly web server was developed, making the model accessible for NP prediction purposes. This approach underscores the effectiveness of NeuroCNN_GNB in leveraging advanced CNN architectures and ensemble learning strategies to increase NP prediction accuracy.

Building on the progress of advanced NP prediction techniques, Akbar et al. presented an automatic and computationally efficient NP predictor.^33^ The same dataset as that of Bin et al.^26^ was collected. Preprocessing steps similar to those in the Bin et al.^26^ trial were applied to determine which sequences to retain or discard. A key aspect of their research was assessing the evolutionary information for amino acid sequences through bigram position-specific scoring matrices (Bi-PSSM), K-spaced bigrams (KSBs), and discrete wavelet transform (DWT). These techniques were integrated to generate highly discriminative vectors for noise reduction, with additional sequential features extracted via one-hot encoding to further enhance the predictive ability of the model. The optimal features are identified via Shapley additive explanations, reducing the dimensionality of the feature vector from 1,140 to 158, and thus lowering the computational cost while retaining essential information. Various classification models and ensemble classification techniques were applied, and to further increase the prediction accuracy, an ensemble learning model was formed. Six different ML models were initially trained, and their predicted labels were combined via a genetic algorithm (GA) to boost the final prediction results. This ensemble approach optimized the overall performance, resulting in the highest prediction accuracy compared with existing models. Among these models, the model developed using XGBoost demonstrated higher performance, achieving an accuracy of 92.55% on independent test data.

## Available databases for computational NP prediction

While several databases contain data related to NPs from different aspects, compiling a comprehensive resource encompassing all would significantly benefit researchers in this field. This section delves into a selection of these data, highlighting their unique contributions to NP prediction methods. Table 4 summarizes the most frequently utilized databases for this purpose.

**Table 4:** Overview of the most frequently used databases for computational NP prediction

	Name	Data content			Description	Open access data	Experimentally validated
		Size	Diversity	Species coverage			
1	NeuroPedia – 2011^56^	847	*Neuropeptides*	*Human, chimpanzee, mouse, rat, bovine, rhesus macaque and California sea*	*NP sequences,* *genomic taxonomic information,* *spectral libraries*	✔	✔
2	NeuroPep – 2015^62^	5949	*Neuropeptides*	*493*	*source organisms,* *tissue specificity,* *families,* *post-translations,* *3D structures (if available),* *literature references*	✔	✔
3	DINer – 2017^60^	4700	*Neuropeptides*	400	NP sequence, physiological functionality, images of receptor-binding sites, families, cladograms, sequence algnments, and logos, standardized nomenclature	✔	✔
4	SwePep – 2006^67^	4180	endogenous peptides	394	*Mass,* *isoelectric point,* *sequence,* *precursor protein*	✔	✔
5	Neuropeptides – 2010^68^	-	neurotransmitters, hormones, and other signaling molecules.	*Primarily focused on human neuropeptides*	*NPs,* *precursors,* *their genes,* *active peptides* *expression in the brain*	✔	✔
6	UniProt – 2005^49,69^	> 570,420 reviewed proteins	*Proteins (UniProtKB)* *Species (Proteomes)* *Protein clusters (UniRef)* *Sequence archive (UniParc)*	Bacteria (387,759) Viruses (109,625) Eukaryota (4,248) Archaea (3,810)	Protein sequences, functional information, cross-references, keywords, post-translational modifications, toxicology data, strain information, protein data bank integration, uniref, digital object identifiers	✔	✔
7	SATPdb – 2016^59^	19192	*antimicrobial, anticancer, antiviral, antibacterial, antifungal, antiparasitic, antihypertensive, cell-cell communication, drug delivery vehicle and toxic*	-	Peptide sequences, structural annotations, therapeutic activities	✔	✔

The SwePep database^67^ is a publicly available resource specifically designed for endogenous peptides. This database facilitates the identification of peptides from complex samples via MS. It compiles information on 4,180 annotated endogenous peptides from 394 diverse species. Each entry provides valuable attributes such as mass, sequence, isoelectric point, and precursor protein information. Notably, SwePep introduced a method for NP identification that significantly accelerates the process within the field of peptidomics. This approach enables researchers to compare experimentally derived peptide masses with those stored in the database. To the best of our knowledge, SwePep stands as the first database specifically dedicated to NPs, representing a foundational step toward fully computational prediction of these crucial signaling molecules.

Additionally, Burbach established the Neuropeptides database,^68^ a comprehensive platform offering detailed annotations, structural classifications, and hyperlinks to related resources. This extensive database not only encompasses gene information but also provides in-depth data on NPs, including detailed precursor information, expression in the brain, and processed active peptide names. The years of its successful application demonstrate the effectiveness and functionality of the data stored in the NP database. Notably, data from this resource have been employed in various non-computational research studies.^69^^,^^70^

Moreover, in 2011, Kim et al. incorporated empirical data through MS into the NeuroPedia database,^56^ a user-friendly web server providing easy access to structured information on NPs. During the period when tandem mass spectrometry (MS/MS) served as the primary technology for high-throughput NP identification, NeuroPedia emerged as a valuable source for NP analysis via MS/MS. This database encompasses NP sequences and genomic taxonomic information, alongside spectral libraries containing identified MS/MS spectra of homologous NPs across diverse classes. The accessibility of the NeuroPedia spectral libraries allows the utilization of spectral library search tools, which are known to increase the sensitivity of peptide identification, further facilitating the identification of NPs. Over the years, researchers have leveraged NeuroPedia’s data-diverse applications. For example, Bojić et al. employed a database for drug-target prediction in neurogenic hypertension and vasovagal syncope.^71^ The impact of Neuropedia extends beyond its role in *in silico* studies, it has also served as a crucial foundation for the development of the NeuroPep^62^ and SATPdb^59^ databases, which is discussed in detail later in this section.

Furthermore, while initially focused on MS techniques, NP databases have evolved to encompass computational applications. In 2015, Wang et al. addressed this need by developing NeuroPep,^62^ an experimentally validated database specifically designed for computational approaches. This comprehensive source provides detailed annotations for each NP, including source organisms, tissue specificity, family, names, post-translational modifications, three-dimensional (3D) structures (when available), and literature references. NeuroPep’s user-friendly interface further enhances its utility by integrating web tools for browsing, sequence alignment, and mapping functionalities. NeuroPep has become a valuable asset for NP prediction studies, with its first documented application being PredNeuroP.^26^ The same research team presented NeuroPep 2.0,^65^ an updated repository containing an extensive collection of 11,417 unique NP entries, representing a significant increase from its earlier version. There is valuable information added in the new version, such as information about NP receptors. There are also 3D structures predicted for NPs lacking experimental data, via advanced computational tools such as AlphaFold2 and APPTEST. Published in 2024, NeuroPep 2.0 has already been leveraged in newly developed prediction algorithms such as NeuroPred-PLM.^32^ NeuroPep 2.0 is freely accessible through its online website to serve as a valuable resource for researchers investigating the therapeutic potential of neuropeptides across various disease contexts.

Additionally, several existing databases not specifically designed for NPs have found application in NP prediction studies. One such example is DINeR,^60^ a comprehensive collection of insect NPs created by Yeoh et al., which is available through a user-friendly web server. DINeR facilitates the search and retrieval of information about 50 well-described NP families across over 400 insect species. This resource provides detailed insect isoform sequences, physiological functionality, and even images of receptor-binding sites. Moreover, DINeR offers extensive data about functions, locations, sequence alignment, and logos for all 50 NP families of insects, making it a valuable tool for researchers. The data from DINeR have been instrumental in the development of NeuroPIpred,^25^ a web application specifically designed for insect NP identification.

Initially, researchers relied on the literature to access *Aplysia* prohormone data, as exemplified by the work of Hummon et al. in 2003.^20^ However, a more efficient approach emerged with the establishment of collaborative research in the Collaborative Research in Computational Neuroscience (CRCNS)^72^ web server. This comprehensive data-sharing platform, initiated by Teeters et al., aimed to promote the publication of high-quality datasets specifically relevant to the field of computational neuroscience. As a centralized resource, CRCNS offers diverse types of information, including dedicated sections focused on *Aplysia* data alongside physiological recordings from sensory and memory systems, and even eye movement data. The platform has facilitated data publication and sharing by researchers.^73^^,^^74^^,^^75^

Furthermore, the universal protein resource (UniProt),^76^ established in 2002, serves as a comprehensive protein resource. This database contains nearly 190 million high-quality protein sequences enriched with detailed functional annotations. UniProt’s extensive data have permeated virtually all aspects of protein sequence analysis, and the field of NP prediction is no exception. Almost all the articles cited within this work utilized UniProt as a source for their model’s input sequences, with a significant subset using UniProt data as a negative dataset.

To further enrich the resources available for NP research, Singh et al. developed the SATPdb.^59^ This database integrates 22 distinct public datasets offering a collection of 19,192 unique experimentally validated therapeutic peptide sequences. These sequences encompass natural, non-natural, and modified amino acids, ranging from 2 to 50 amino acids in length. The curated and annotated data in SATPdb have significantly impacted research in drug discovery, peptide engineering, and therapeutic peptide design, serving as valuable resources for *in silico* and ML research. The data from SATPdb have been employed in various computational studies as a component of the training dataset, with a majority focusing on predicting therapeutic peptides, including anticancer peptides, antidiabetic peptides, and antimicrobial peptides. As demonstrated in Table 3, the construction of NeuroPIpred^25^ also leveraged a portion of its positive data from SATPdb.

## Computational tools utilized for NP prediction

Over the years, many computational tools have been developed to facilitate bioinformatics processes and make these experiments less time-consuming and costly. This work reviews several tools developed for predicting different aspects of NPs. This section provides an in-depth exploration of tools utilized in NP prediction research across various phases of development.

Determining the similarity between genome and protein sequences is crucial in bioinformatics for various applications, including NP prediction.^77^^,^^78^ Two main categories of sequence comparison methods exist, alignment-based methods and alignment-free methods. Although alignment-based methods provide detailed information, these can be time-consuming and computationally expensive. As a result, faster alignment-free approaches have been developed.^78^

This section discusses several bioinformatics tools with diverse functionalities for sequence comparison, which are often employed in the context of NP prediction.

CD-HIT, one of the first tools developed for sequence clustering and redundancy reduction, utilizes a combination of sequence alignments and a greedy incremental algorithm for efficient clustering.^79^^,^^80^^,^^81^^,^^82^ Over time, CD-HIT has evolved, offering specialized tools such as cd-hit-est, cd-hit-2d, and cd-hit-est-2d, each catering to specific tasks such as comparing protein and nucleotide sequences into distinct datasets.^83^ These advancements have transformed CD-HIT into a robust software suite. In the context of NP prediction, where sequence-based approaches are prevalent, reducing redundancy can improve prediction accuracy. Therefore, CD-HIT is often employed as an initial pre-processing step in many NP prediction studies.

Additionally, Kr,^84^ developed by Domazet-Lošo and Haubold, utilizes alignment-free methods to compare sequences and calculate pairwise distances between genomes. Like CD-HIT, Kr has undergone improvements and is available as Kr version 2. While effective, Kr has documented limitations, including high computational resource requirements^85^ and lower accuracy,^86^ which led to the development of the ALFY^87^ by the same researchers. Both Kr^84^ and ALFY^87^ are compatible with Linux and Apple Macintosh operating systems.

Moreover, the pursuit of faster and more accurate methods led to the introduction of andi in 2015 by Haubold et al.^88^ Compared with Kr,^84^ andi^88^ demonstrated significant improvements in accuracy, speed, and memory efficiency for estimating evolutionary distances, particularly among closely related genomes. However, the demand for advancement continues. In 2019, Farkaš et al. introduced SWSPM,^89^ an alignment-free DNA comparison method based on a signal-processing approach. The authors conducted extensive comparisons between SWSPM^89^ and existing methods such as Kr^84^ and andi^88^ via several databases. Their findings revealed that SWSPM^89^ outperforms both in terms of accuracy, run time, and memory requirements.

Furthermore, identifying open-reading frames (ORFs) in genomes helps us understand potential protein-coding regions. By definition, an ORF is a continuous sequence of codons (DNA triples) that begins with a start codon and ends with a stop codon, exceeding the minimum length.^90^ Several web-based ORF-finding tools have been developed to facilitate the efficient extraction of ORFs from DNA sequences. This section explores a selection of these tools and their functionalities. The NCBI ORF-finder^91^ web tool, offered by the National Center for Biotechnology Information, allows users to submit DNA sequences and search for potential ORFs. It provides various options, including adjustable minimum ORF length, selection of genetic code for translation, and display of protein translation. The NCBI ORF-finder^91^ is the most commonly used web server for this purpose. It provides a user-friendly interface and a range of extra functionalities such as a SMART BLAST option, enabling users to validate proteins they found within their target genome. Moreover, the tool incorporates a horizontal bar display that demonstrates the identified ORFs, along with their respective sizes, and any overlapping regions across the gene sequence.

Additionally, the sequence manipulation suite (SMS)^92^ is a collection of online bioinformatics tools offering a wide range of functionalities for DNA and sequence analysis, including generating, manipulating, formatting, and interpreting sequences. Moreover, SMS provides an option to host each of its software tools on its website, an option utilized by web tools such as Gene INFINITY.^93^

The EMBOSS Suite^94^ also provides an ORF-finder web server called “getorf” that locates and outputs the sequences of ORFs in nucleotide sequences. There is an option in EMBOSS^94^ and NCBI ORF-finder^91^ in which users can choose to obtain output in nucleotide sequences or protein translation of the ORFs (peptide sequences).

Identifying ORFs within genes can provide valuable information about their potential for NP synthesis and functionality. This information helps researchers understand the origins and activities of NPs within the neural and immune systems.^95^

Furthermore, the accurate identification of protein localization across various cellular compartments is fundamental for functional annotation in biological systems. In addition to its role in elucidating protein function, precise localization prediction serves as a crucial step in identifying potential drug targets^96^ and unraveling the underlying mechanisms of diseases associated with aberrant subcellular distribution.^97^ For this purpose, the first version of DeepLoc^98^ was developed in 2017 by Almagro Armenteros et al., which classifies eukaryotic proteins into their respective subcellular localizations and membrane associations via DL techniques. The latest version of this tool, DeepLoc 2.1,^99^ utilizes DL techniques, specifically pre-trained transformer-based protein language models. Owing to its enhanced accuracy and accessibility through the web server, DeepLoc 2.1^99^ is a valuable resource for researchers studying protein localization and cellular processes.

## Discussion

### NP prediction models

**Figure 3:**
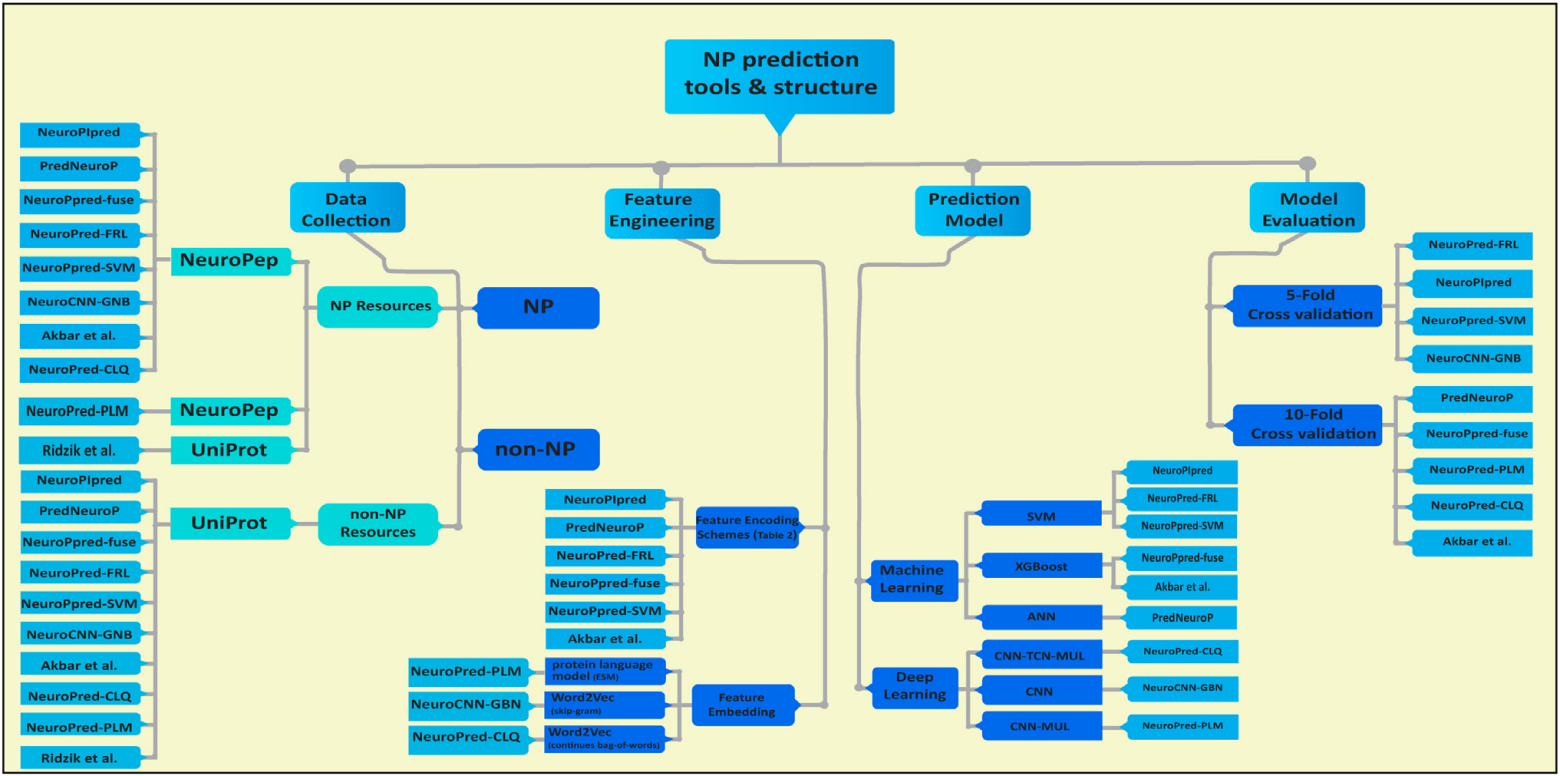
Summary of methods in NP prediction stages An overview of the methods used by different NP prediction tools at each stage of prediction, including data collection, feature extraction, model types, and model evaluation techniques. The diagram organizes tools by stage to provide a clear comparison of methodological approaches.

NPs function as neurotransmitters and play a critical role in the immune system, making them attractive targets for drug discovery. This review explores the evolution of NP prediction models over the past 2 decades, with a focus on methodologies, datasets, and key findings. Figure 3 illustrates the comprehensive workflow for NP prediction, which is segmented into four critical stages: data collection, feature engineering, the ML/DL model, and model evaluation.

Initially, the process involves collecting both positive and negative NP data from various sources. The sources for positive data have evolved over time. Before the NeuroPep^62^ database was published in 2015, researchers such as Ridzik and Brejová^57^ utilized data from Swiss-Prot^49^ for their NP prediction model because of the absence of any database dedicated to the NPs. However, after the publication of NeuroPep,^62^ it became the primary resource for NP prediction models until the introduction of NeuroPep 2.0^65^ in 2024. For the negative dataset, Swiss-Prot has remained the sole resource.

The subsequent stage, feature engineering, focuses on embedding and encoding features via diverse techniques. In this stage, DL techniques employ feature embedding strategies such as BERT-cls or word2vec (Table 2), whereas ML techniques use feature encoding schemes, the complete list of which is illustrated in Table 1. These features are then fed into the ML or DL models. The most commonly used algorithm is the SVM, which has demonstrated superior performance compared with other ML algorithms. The final stage involves rigorous model evaluation via 5- and 10-fold cross-validation methods, ensuring robust and reliable predictions.

In recent advancements in NP prediction methodologies, various models have been developed to increase prediction accuracy and computational efficiency. The development of NP prediction software began in 2014, when Ridzik and Brejová initiated efforts to identify NPs.^57^ Challenges in identifying precursor proteins, predicting cleavage sites, and annotating NPs derived from these precursors were addressed. Two models were introduced: an SVM model for predicting cleavage sites and a semi-CRF model for annotating NP sequences. Their cleavage site predictor achieved a classification accuracy of 91.1%. Additionally, the NP annotation model, which clustered and filtered protein sequences, reached a final accuracy of 79.1% after integration with the cleavage site predictor.

This research utilized a unique approach, distinct from other NP prediction methods discussed in this article, by developing and combining two distinct models to annotate the NP sequences. The other NP prediction tools developed subsequently have not been as complex. The following is a summary of these tools.

(1) Agrawal et al. developed NeuroPIpred, a web application that uses various feature selection schemes and ML models to predict insect NPs. The accuracies reported by the authors were 83.71% and 97.47% for their datasets.^25^ Similarly, PredNeuroP, developed by Bin et al., was designed on the basis of a two-layer stacking model with multiple ML algorithms and feature encoding schemes. The authors reported an accuracy of 87.2% for their model.^26^
(2) In 2021, two NP prediction models were published. One of them, NeuroPred-FRL, was developed by Hasan et al. and employs FRL. By training 66 models that combined various feature encoding schemes and ML algorithms, the highest performance was achieved using binary encoding and the SVM algorithm, with an accuracy of 91.9%.^27^ The second model, NeuroPpred-Fuse, developed by Jiang et al., is an interpretable stacking model with feature selection algorithms. By fusing the encoded features, aggregating the results from three base learners, and using a meta-learner, the NeuroPpred-Fuse achieved an accuracy of 90.6%.^28^
(3) The following year, Chen et al. developed NeuroPred-CLQ, a DL model that combines word2vec embeddings and a hybrid CNN-TCN multihead attention architecture. This approach effectively integrates the strengths of various neural network architectures, achieving an accuracy of 93.6%.^29^
(4) In 2023, Liu et al. introduced NeuroPpred-SVM, a web server utilizing a two-layer ML model and diverse feature encodings, achieving a commendable accuracy of 91.5% through the integration of SVM and logistic regression algorithms.^31^ In the same year, Wang et al. developed NeuroPred-PLM, leveraging a 12-layer protein language model (ESM) combined with a multiscale CNN, which demonstrated an average accuracy of 92.7%.^32^
(5) Continuing this trend, Liu et al. proposed NeuroCNN_GNB, a two-layered stacking model utilizing CNNs and ensemble learning, achieving a high accuracy of 91% through the integration of Skip-Gram embeddings and a Gaussian naive Bayes classifier.^30^ Similarly, Akbar et al. presented a computationally efficient NP predictor utilizing the Bi-PSSM, KSB, and DWT for evolutionary information assessment. Their model achieved an accuracy of 92.55% with XGBoost.^33^

These studies collectively demonstrate significant advancements in NP prediction, introducing innovative methodologies to enhance model performance and computational efficiency. The reported accuracy across these tools ranges from 83% to 93%, with NeuroPIpred notably achieving 97.47% accuracy for their NeuroPIpred_DS2 dataset of C-terminal amidated NPs.^25^ However, according to the comparison tables provided by Chen et al.,^29^ Akbar et al.,^33^ and Jiang et al.,^28^ NeuroPIpred^25^ reported a lower accuracy of 53.6%. This discrepancy can be attributed to the fact that NeuroPIpred^25^ trained on different databases. Unlike others, it uses DINeR^60^ instead of NeuroPep^62^ for its positive data.

Almost all of the NP prediction models rely on mutual positive and negative datasets extracted from NeuroPep^62^ and UniProt^49^ by Bin et al. in 2020.^26^ In terms of the most accurate models, NeuroPred-CLQ,^29^ NeuroPred-PLM,^32^ and the NP prediction tool of Akbar et al.^33^ reported accuracies greater than 92%. Among these, only the model of Akbar et al. utilized feature encoding schemes (Table 1) for feature extraction. The primary advantage of the NeuroPred-PLM model can be attributed to the use of NeuroPep 2.0.^65^ While training models with more data generally lead to better performance, NeuroPred-CLQ achieves higher accuracy using the previous dataset. Since NeuroPred-PLM and NeuroPred-CLQ do not use the same database for training and test processes, comparisons of these models face limitations. However, generally, a combination of CNNs can increase the accuracy of the model to 92.2% and 93.6% for NeuroPred-PLM and NeuroPred-CLQ, respectively.

The Akbar et al. NP prediction model stands out due to the use of Bi-PSSM, KSB, and DWT to assess the information in amino acid sequences. Since Bi-PSSM and KSB are feature encoding schemes specifically designed for protein sequences, and DWT is utilized for noise reduction, the model achieved an accuracy of 92.5% without relying on extensive data from NeuroPep 2.0 or a combination of hybrid models. This accuracy was achieved solely with an XGBoost model. Subsequent advancements in NP prediction methodologies have been marked by the adoption of data-driven strategies, such as word2vec,^29^^,^^30^^,^^32^ which have led to substantial improvements in prediction accuracy.

In Figure 3, the methods used by NP prediction tools are summarized in several crucial steps, including data collection, feature engineering, ML/DL model construction, and model evaluation. The first step is data collection and preprocessing, revealing that almost all the models were trained with the same data. This highlights the crucial need for high-quality and experimentally validated data to ensure the reliability and credibility of these models. The scarcity of such data underscores the importance of sourcing new and diverse datasets, as this directly impacts model performance. Figure 3 shows that UniProt was utilized as a positive data resource by Ridzik and Brejová in 2014, and the primary choice for the tools developed since 2015 to extract positive data has been NeuroPep, whereas UniProt has been the sole resource for negative data in all the developed tools.

Following data collection, feature engineering plays a significant role in improving prediction accuracy. This stage involves various techniques such as feature extraction, selection, optimization, embedding, and encoding. These techniques aim to extract and optimize the features used for prediction. This ultimately leads to better model performance. As demonstrated in Figure 3, methods were categorized into two types: studies using feature embedding methods (Table 2) and studies utilizing feature encoding schemes (Table 1) to extract features from their data. Feature encoding schemes are methods used to transform biological sequences, such as protein or nucleotide sequences, into numerical representations that can be processed by ML or DL models. These encoded features capture various properties and patterns in the sequences, enabling the models to make predictions or classifications. This transformation is performed on predefined schemes that often rely on the physical, chemical, or positional properties of amino acids or nucleotides.^100^ However, feature embeddings are defined as continuous vector representations learned from the data. These embeddings capture more complex and context-dependent information about sequences. They are generally more powerful but can be harder to interpret. Extracting complex relationships, these methods are suitable for more sophisticated models and larger datasets.^101^

In NP prediction, various tools utilize a diverse array of feature encoding schemes to increase accuracy and reliability. The tools NeuroPIpred,^25^ PredNeuroP,^26^ NeuroPpred-Fuse,^28^ NeuroPred-FRL,^27^ NeuroPpred-SVM,^31^ and the method proposed by Akbar et al.^33^ demonstrate distinct approaches by employing both basic and advanced feature encoding strategies. Basic composition-based schemes such as amino acid composition, dipeptide composition, and tripeptide composition are commonly used by NeuroPIpred,^25^ PredNeuroP,^26^ and NeuroPpred-Fuse,^28^ highlighting their foundational role in peptide analysis. Advanced composition-based schemes, including the profile-based composition of amino acids, k-gap composition, and quasi-sequence order, are integrated into more sophisticated tools such as NeuroPpred-Fuse^28^ and NeuroPred-FRL,^27^ offering deeper insights into sequence characteristics. Binary encoding schemes such as binary profiles and binary encoding, utilized by NeuroPIpred^25^ and NeuroPpred-Fuse,^28^ provide a straightforward yet effective method of feature representation. Statistical and information-based schemes, such as amino acid entropy and composition-transition distribution, which are employed by tools such as PredNeuroP,^26^ capture the statistical properties and distributional attributes of amino acid sequences. Specialized schemes, including the amino acid pair scale and DWTs, further exemplify the diverse range of feature encoding schemes, underscore the multifaceted nature of NP prediction, and highlight the continuous evolution of computational techniques in bioinformatics.

Similar feature encoding schemes, such as composition-based methods, which have been employed in studies,^25^^,^^26^^,^^27^^,^^28^^,^^31^^,^^56^ were used in early NP prediction models. Despite limitations in capturing the impact of individual amino acid positions within sequences, these methods achieve remarkable accuracy in the range of 90%–92%. However, Akbar et al. recently demonstrated that it is possible to achieve an accuracy of 92.50% by utilizing feature encoding schemes and an ML model.^33^

In conclusion, feature encoding and feature embeddings are both methods used to represent biological sequences in a numerical format suitable for the ML and DL models; however, they have different approaches and capture different kinds of information. Although combining both methods can sometimes yield improved results, the research by Liu et al. in developing NeuroCNN_GNB^30^ applied four types of feature extraction methods, including one-hot encoding, AAIndex, G-gap dipeptide encoding, and word2vec. In their research, the model using the embedding approach, word2vec, outperformed the models based on encoding technique.

The next two steps demonstrated in Figure 3 involve developing and evaluating the NP prediction models. In early NP prediction models, SVM algorithms were the preferred choice because of their effectiveness in classification tasks and superior performance. However, in the research conducted by Akbar et al., all the applied learners, including SVM, AdaBoost, and XGBoost, achieved the best performance with their XGBoost models. In addition, the CNN models are the most popular DL models among the NP prediction tools. However, better performance is observed in NeuroPred-CLQ when the CNN is combined with a TCN and a multihead-attention model. Furthermore, to assess the performance of the chosen model, considering the available computer resources, k-fold cross-validation emerges as a prevalent strategy.

### Databases

The completion of the Human Genome Project led to a surge in available genome sequences, fueling the development of diverse databases. Databases such as ACD,^102^ SATPdb,^59^ and HERB^103^ serve specific purposes, focusing on sharing therapeutic peptides. The SEGUID database^104^ provides unique protein sequence identifiers and is a valuable source for proteome-wide research. Meanwhile, dedicated databases specially designed for computational NP research are still under development. NeuroPep,^62^ introduced in 2015, quickly became the go-to resource for training NP prediction models.^26^^,^^27^^,^^28^^,^^31^ In 2024, NeuroPep 2.0^65^ was released, nearly doubling the data size, highlighting the ongoing need for even larger and more specialized databases to advance NP prediction research.

Databases containing NP data are not limited to NP prediction research. For example, Grønning et al. utilized data from sources such as NeuroPedia, SATPdb, and various public databases to develop MultiPep,^105^ a DL-based classifier for peptide clustering based on bioactivity. These findings demonstrate the potential of these databases in supporting the development of tools such as MultiPep,^105^ which could contribute to the advancement of peptide-based therapies.^56^

In this research, the most commonly used databases for training NP prediction ML/DL models are highlighted. Some databases, such as NeuroPep^62^^,^^65^ and DINer^60^ provide direct resources for NPs, whereas others, such as SATPdb^59^ or UniProt,^76^ serve as broader repositories for extracting both NP and non-NP sequences. Further details about these databases are available in Table 4.

### Tools

The study also reviewed bioinformatics tools utilized for data pre-processing and analysis in NP prediction research. Different tools that cater to diverse data types and user needs have been developed. These tools offer services such as clustering, quality validation, sequence alignment, and annotation, often utilizing various methods and performance levels. While some specific tools excel in certain areas, others stand for user-friendliness and accessibility. For example, while powerful clustering methods such as UCLUST^106^ exist, the open-source nature and user-friendly web interface of CD-HIT have made it a preferred choice among researchers.

There might be more peptide-related bioinformatic tools that could benefit NP research, as discussed in the review article by Peng et al.^107^ However, this article focuses on tools already utilized in NP prediction research.

## Conclusion

The evaluation of the NP prediction model over the past decade has demonstrated significant advancements in accuracy and computational efficiency. Models such as NeuroPred-CLQ^29^ and NeuroPred-PLM,^32^ which leverage CNNs and multihead attention layers, have underscored the effectiveness of integrating sophisticated architectures for NP prediction.

Looking forward, the field would benefit from exploring alternative approaches for feature engineering, especially data-driven methods that can enhance the capture of complex sequence patterns. Despite the effectiveness of feature encoding schemes, their limitations in capturing positional information and the nuanced relationships between amino acids highlight the ongoing need for innovation in this area.^19^

The introduction of NeuroPep 2.0,^65^ which incorporates unnatural amino acids, poses intriguing questions about its impact on model performance and computational efficiency. While previous studies, such as the exclusion of such sequences by Bin et al. suggest caution, further investigations are warranted to fully understand the potential benefits or drawbacks of including unnatural amino acids in NP prediction models.

In conclusion, the continued development of NP prediction methodologies, coupled with advanced techniques, promise to enhance further our ability to predict and understand NPs. Addressing these challenges will be crucial in advancing both basic research and the particular applications of NP prediction in drug discovery and therapeutic development.

## Acknowledgments

This article is part of a master’s thesis project conducted at the School of Management and Medical Informatics, Tabriz University of Medical Science, Tabriz, Iran. The research was approved and supported by the 10.13039/501100021771Student Research Committee (SRC) at 10.13039/501100004366Tabriz University of Medical Sciences (74841).

## Author contributions

Conceptualization, R.R. Methodology, L.R.K. Investigation, R.R. Data curation, R.R. Formal analysis, R.R. Writing – original draft, R.R. Writing – review & editing, L.R.K. and R.F. Supervision, L.R.K. and R.F. Resources, R.F. Project administration, R.F.

## Declaration of interests

The authors declare no competing interests.
